# Navigating stress: workplace resilience and mental health nursing in Egyptian psychiatric hospitals

**DOI:** 10.1186/s12912-026-04434-0

**Published:** 2026-03-11

**Authors:** Gellan K. Ahmed, Naglaa Abd El-Megied, Maria Adel Thabet Azer, Doaa Mazen Abdel-Salam, Azza Mohamed Abd El-Aziz

**Affiliations:** 1 Department of Neurology and Psychiatry, Faculty of Medicine, Assiut University, Assiut, Egypt; 2 Department of Psychiatric and Mental Health Nursing, Faculty of Nursing, Assiut University, Assiut, Egypt; 3 Faculty of Nursing, Badr University (BUA), New Nasser City, West of Assiut, Assiut, Egypt; 4 Mental Health Hospital, Ministry of Health, Assiut Governorate, Assiut, Egypt; 5 Department of Public Health and Community Medicine, Faculty of Medicine, Assiut University, Assiut, Egypt

**Keywords:** Psychological well-being, Workplace resilience, Mental health nurses, Psychiatric nursing, Egypt

## Abstract

**Background:**

Mental health nurses encounter distinctive occupational challenges that significantly influence their psychological well-being and resilience capacity. These challenges include managing patients with complex mental health conditions, navigating high-stress clinical environments, and addressing the substantial emotional demands inherent in psychiatric nursing practice. This study aimed to assess the psychological well-being and workplace resilience of nurses employed in mental health hospitals in Upper Egypt and to identify factors associated with these outcomes.

**Methods:**

A cross-sectional study was conducted among 200 mental health nurses in mental health hospitals located in Assiut district, Upper Egypt. Data were collected through semi-structured questionnaires administered via direct interviews with study participants. Psychological well-being was measured using Ryff’s Psychological Well-Being Scale, while workplace resilience was assessed using the Resilience at Work Scale.

**Results:**

The overall mean psychological well-being score was 4.32 ± 0.46. Among the psychological well-being dimensions, personal growth demonstrated the highest mean score (4.58 ± 0.81). The overall mean resilience at work score was 4.60 ± 0.56. Cooperative interaction exhibited the highest mean score (5.48 ± 0.54), whereas maintaining perspective showed the lowest mean score (3.67 ± 1.00). Significant predictors of both psychological well-being and workplace resilience included employment in university hospitals and higher socioeconomic status. Male gender emerged as a significant predictor of psychological well-being.

**Conclusions:**

This study demonstrates that a substantial proportion of mental health nurses exhibit high levels of workplace resilience, while the majority demonstrate moderate levels of psychological well-being. Strengthening workplace resilience represents a critical strategy for healthcare organizations seeking to support the psychological well-being of mental health nursing staff. The results emphasize the essential role of organizational support and structured interventions in fostering workplace resilience and safeguarding the psychological well-being of mental health nurses.

## Introduction

Nurses employed in psychiatric institutions experience considerable emotional distress due to workplace adversity stemming from emotionally challenging situations and complex interpersonal demands [1]. These nurses regularly engage with patients exhibiting confrontational behaviors, including suicidal ideation, aggression, and bullying [2]. Sustained exposure to emotional stress can adversely affect nurses’ well-being, potentially resulting in mental distress, burnout, and compassion fatigue [3]. Psychological well-being is conceptualized as a state of optimal positive psychological functioning that enhances an individual’s capabilities and fosters adaptive capacity to overcome challenges and obstacles over time [4]. This construct encompasses six dimensions of positive psychological functioning: autonomy, purpose in life, personal growth and development, self-acceptance, environmental mastery, and positive relationships with others [5, 6]. The literature provides mounting evidence of workplace stress’s detrimental effects on the psychological well-being of mental health nurses, including elevated levels of burnout, fatigue, depression, and anxiety [7–9].

Emerging evidence suggests that enhancing workplace resilience may help mitigate the adverse effects of emotional adversity on nurses’ psychological well-being and clinical practice [10]. Strengthening workplace resilience among mental health nurses can buffer the negative impact of occupational stress on their psychological well-being [1]. Workplace resilience is defined as a dynamic adaptation process following distress that promotes enhanced psychological well-being and improved work performance [11].

In Egypt, although numerous studies have examined burnout [12, 13], stress [14, 15], anxiety [15, 16], and depression [15, 17] among nurses across various healthcare settings, few have investigated psychological well-being and resilience among mental health nurses specifically. This issue is particularly important in the Egyptian context, where mental health nurses work within resource-constrained healthcare systems and face high occupational demands, underscoring the urgent need for organizational support and structured intervention programs to strengthen workplace resilience and protect psychological well-being.

Despite growing international evidence on psychological well-being and workplace resilience among nurses, empirical data focusing specifically on mental health nurses in Egypt remain scarce. Existing national research has largely concentrated on burnout, stress, anxiety, and depression across general nursing populations, with limited attention to psychological well-being and resilience within psychiatric care settings.

Therefore, this study was conducted to assess the psychological well-being and workplace resilience of nurses employed in mental health hospitals in Assiut District, Upper Egypt, and to investigate factors associated with these outcomes. To the best of the authors’ knowledge, this study is the first to comprehensively examine psychological well-being alongside workplace resilience among mental health nurses in psychiatric hospitals in Egypt. This study will provide novel, context-specific evidence that addresses a critical gap in the literature.

## Methods

### Study setting and design

A cross-sectional study was conducted in mental health hospitals located in Assiut district, Upper Egypt. Two mental health hospitals operate in Assiut District: one administered by the Ministry of Health and the other affiliated with university hospitals. These two governmental mental health hospitals were selected as the study setting because they represent the primary providers of psychiatric care for a large population in Upper Egypt. These hospitals serve patients from both urban and rural areas across several governorates and operate under different administrative systems (Ministry of Health and university-affiliated hospitals), allowing comparison across distinct organizational contexts. The study population consisted of staff nurses working in these two governmental mental health hospitals located in Assiut District, Upper Egypt.

### Sampling

The sample size was determined using Epi Info software, based on the following parameters: a hypothesized proportion of 50%, a 95% confidence level, an acceptable margin of error set at 5%, and a design effect of 1. Sample size was 384 nurses. Since the total number of nurses in the two hospitals are 220. So, the authors calculate adjusted sample size:

$$\:{n}_{adjusted}=\frac{n}{1+\frac{n-1}{N}}=\frac{384}{1+\frac{383}{220}}\approx\:140$$

To account for potential non-response and incomplete data and to improve the precision and power of the study, the final sample size was increased to 200 nurses. A convenience sampling method was employed in this study, including staff nurses working in the two mental health hospitals in Assiut District who met the inclusion criteria. The study sample comprised 52 staff nurses from the mental health hospital (Ministry of Health facility) and 148 staff nurses from Assiut University Hospital for Psychiatry.

Convenience sampling was adopted in the present study due to the limited number of mental health nurses, variability in work shifts, and the need to ensure feasibility of data collection within the constrained clinical settings of psychiatric hospitals.

Nurses who provided informed consent to participate and possessed more than one year of clinical experience were included in the study. Nurses who did not fulfill the aforementioned criteria were excluded.

### Data collection instruments

Data was collected through structured questionnaire completed via direct interviews with study participants. The study was conducted in six months duration from the mid of December 2024 till the end of May 2025. All interviews were conducted by trained researchers with academic backgrounds in mental health nursing. They ensure that all interviewers understand the questions and conduct mock interviews to identify unclear questions and improve consistency. The questionnaire consisted of four sections:

**Section One** elicited sociodemographic data including age (years), gender, residence, educational level, religion, marital status, years of clinical experience, and workplace setting.

**Section Two** employed the socioeconomic status scale developed by El-Gilany et al., 2012 [18]. This instrument comprises seven domains: education and culture, occupation, family characteristics, family possessions, economics, home sanitation, and healthcare access. The scale yields a total score of 84, with socioeconomic status levels categorized as follows: <42 = very low socioeconomic status; 42-<63 = low socioeconomic status; 63-<71.4 = middle socioeconomic status; and 71.4–84 = high socioeconomic status. Strong correlations exist among most of the seven domains. The scale demonstrated borderline acceptable internal consistency (Cronbach’s α = 0.66) [18].

**Section Three** utilized Ryff’s Psychological Well-Being Scale, developed by Ryff & Keyes, 1995 [5]. This 18-item instrument includes three items for each of six well-being dimensions: self-acceptance, autonomy, environmental mastery, purpose in life, positive relations with others, and personal growth. Participants rated each item’s applicability to themselves using a 6-point Likert scale ranging from ‘strongly disagree’ (1) to ‘strongly agree’ (6). Total scores range from 18 to 108, with psychological well-being levels classified as follows: 18–48 = poor; 49–78 = moderate; and ≥ 79 = favorable. The original scale demonstrated high reliability (Cronbach’s α = 0.89) and established content validity [19].

**Section Four** incorporated the Resilience at Work Scale developed by Winwood et al., 2013 [20]. This 20-item instrument comprises seven components measured on a 7-point Likert scale (0–6), ranging from 0 = strongly disagree to 6 = strongly agree. The seven components are: Living Authentically (three items), Finding One’s Calling (four items), Maintaining Perspective (three items), Managing Stress (four items), Interacting Cooperatively (two items), Staying Healthy (two items), and Building Networks (two items). Total scores were calculated to derive a composite resilience value. Resilience at work levels were determined using mean scores, with participants scoring below 61, 61–81, and above 81 classified as having low, moderate, and high levels of workplace resilience, respectively. This assessment tool demonstrated high internal consistency (Cronbach’s α = 0.89) [20].

Using accepted guidelines for translation back translation, Ryff’s Psychological Well-Being Scale and the Resilience at Work Scale were translated into Arabic. They were back translated into English by a bilingual consultant, Then, the questionnaires faced validity by three expert opinions with no major modifications. To ensure reliability within the present sample, internal consistency was reassessed during conduction of a pilot study among 20 mental health nurses. The results indicated satisfactory reliability, with Cronbach’s alpha coefficients of 0.653 for Ryff’s Psychological Well-Being Scale and 0.790 for the Resilience at Work Scale. These values are considered acceptable for multidimensional psychological constructs in field studies.

### Statistical analysis

Statistical analysis was performed using SPSS version 26. Descriptive statistics for quantitative data included means and standard deviations (SD), while qualitative categorical data were presented as frequencies and percentages. The normality of continuous variables was assessed using the Shapiro–Wilk test. The data was normally distributed. Multivariate linear regression analysis was employed to identify significant determinants of psychological well-being and resilience. Statistical significance was set at *p* < 0.05.

### Ethical considerations

The research proposal was approved by the Ethical Committee at the Faculty of Nursing, at Assiut University (ID approval: 1120230733). The researchers conducted this research in accordance with the principles of the Declaration of Helsinki. Participants had the right to withdraw from the study without any rational at any time. Informed written consent to participate in the study was obtained from nurses after explanation of the study purpose. Privacy and confidentiality were assured.

## Results

Table 1 depicted the sociodemographic characteristics of study participants. The mean age of participants was 37.5 ± 8.8 years. Most participants were female (63.5%), resided in urban areas (60.5%), and were married (83.5%). Nearly half had secondary education (48.5%), and the majority identified as Muslim (85%). The distribution of clinical experience among nurses was as follows: 36.5% had over 20 years, 29% had 11–20 years, 16.5% had 6–10 years, and 18% had 1–5 years. Regarding workplace setting, 74% were employed at Assiut University Hospital for Psychiatry, while 26% worked at the Ministry of Health mental health hospital.

**Table 1:** Personal characteristics of mental health nurses

Sociodemographic characteristics	No. (200)	%
**Age: (years)**		
< 40	129	64.5%
≥ 40	71	35.5%
Mean ± SD (Range)	37.48 ± 8.76 (26.0–59.0)	
**Gender**:		
Male	73	36.5%
Female	127	63.5%
**Residence**:		
Urban	121	60.5%
Rural	79	39.5%
**Educational level**:		
Secondary	97	48.5%
Technical institute	57	28.5%
University or above	46	23.0%
**Religion**:		
Muslim	170	85.0%
Christian	30	15.0%
**Marital status**:		
Married	167	83.5%
Single	22	11.0%
Divorced/ widowed	11	5.5%
**Years of experience**:		
< 10	63	31.5%
10–20	64	32.0%
> 20	73	36.5%
**Place of work**:		
Assiut University Hospital for Psychiatry	52	26.0%
Mental Health Hospital in Assiut District	148	74.0%
**Social level**:		
Very low	45	22.5%
Low	50	25.0%
Middle	49	24.5%
High	56	28.0%

Socioeconomic status assessment indicated that 28% of participants reported high socioeconomic status, 24.5% had middle socioeconomic status, and 47.5% belongs to low or very low socioeconomic status.

### Psychological well-being assessment

**Fig. 1:**
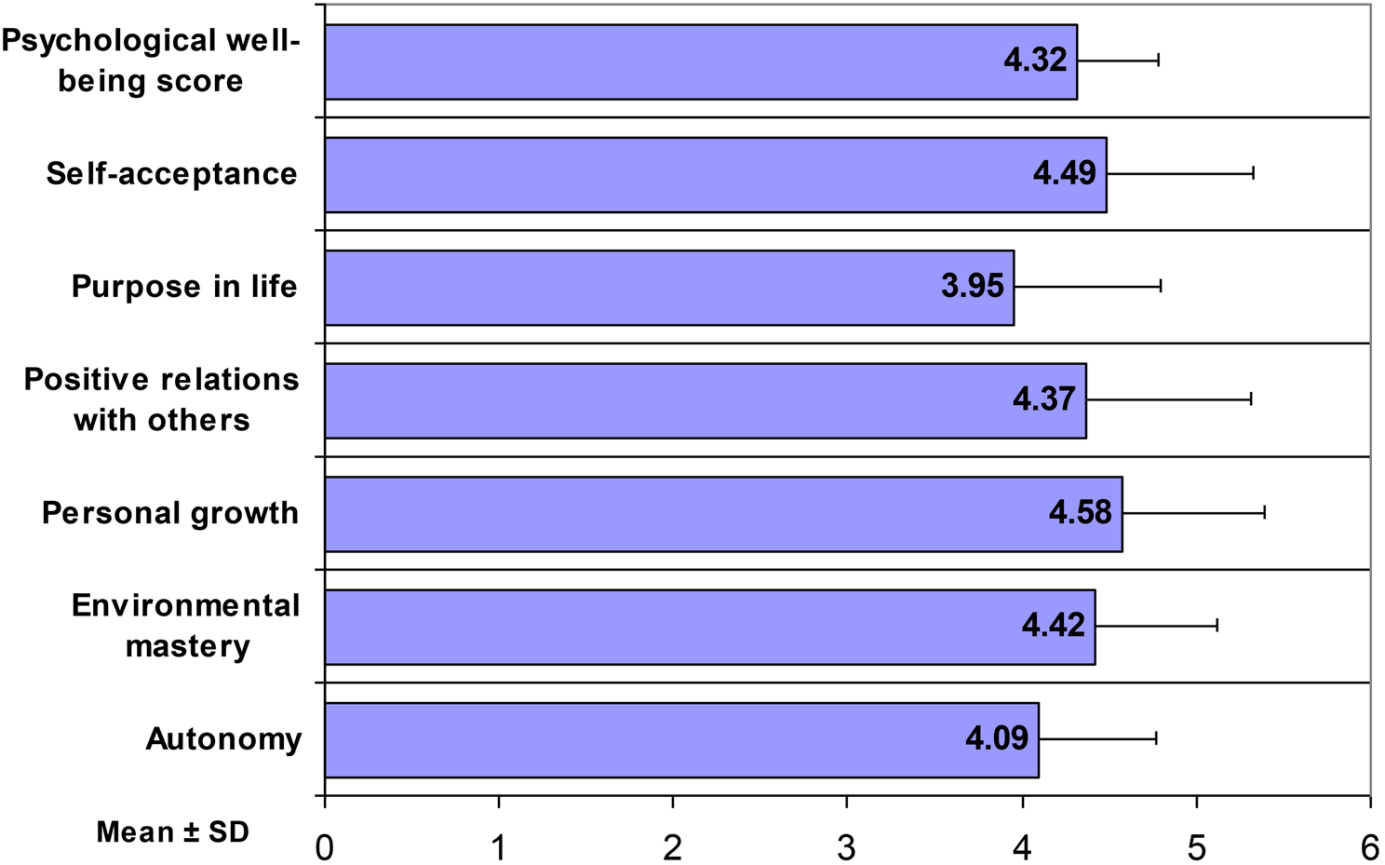
Psychological well-being scores of mental health nurses across several dimensions

Figure 1 illustrates the mean scores for psychological well-being across its six constituent dimensions. The average psychological well-being score was 4.32 ± 0.46 on a 6-point scale, suggesting a moderate level of well-being. Analysis of individual dimensions revealed considerable variation. Personal growth emerged as the dimension with the highest mean score (4.58 ± 0.81), reflecting participants’ perceptions of continued development and self-improvement. This was followed closely by self-acceptance (4.49 ± 0.83). Environmental mastery demonstrated a mean score of 4.42 ± 0.70.

Positive relations with others yielded a mean score of 4.27 ± 0.80, while autonomy demonstrated a mean score of 4.14 ± 0.81, indicating moderate levels of self-determination and independence. Notably, purpose in life exhibited the lowest mean score among all dimensions (3.95 ± 0.85).

**Fig. 2:**
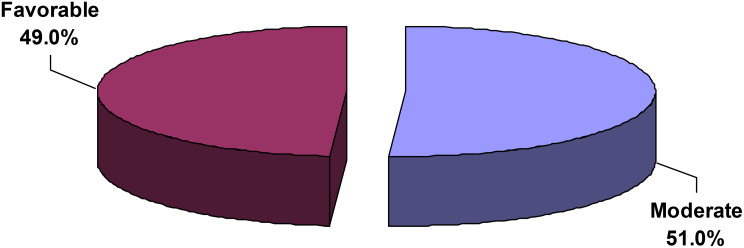
Level of psychological well-being among mental health nurses

Figure 2 demonstrates the distribution of psychological well-being levels across the sample. According to predefined thresholds set by the scale, analysis revealed that 51.0% of nurses exhibited moderate levels of psychological well-being (scores 49–78), while 49.0% demonstrated favorable levels (scores ≥ 79). Notably, no participants fell within the poor psychological well-being category (scores 18–48), indicating that all nurses maintained at least moderate levels of psychological functioning.

### Workplace resilience assessment

**Fig. 3:**
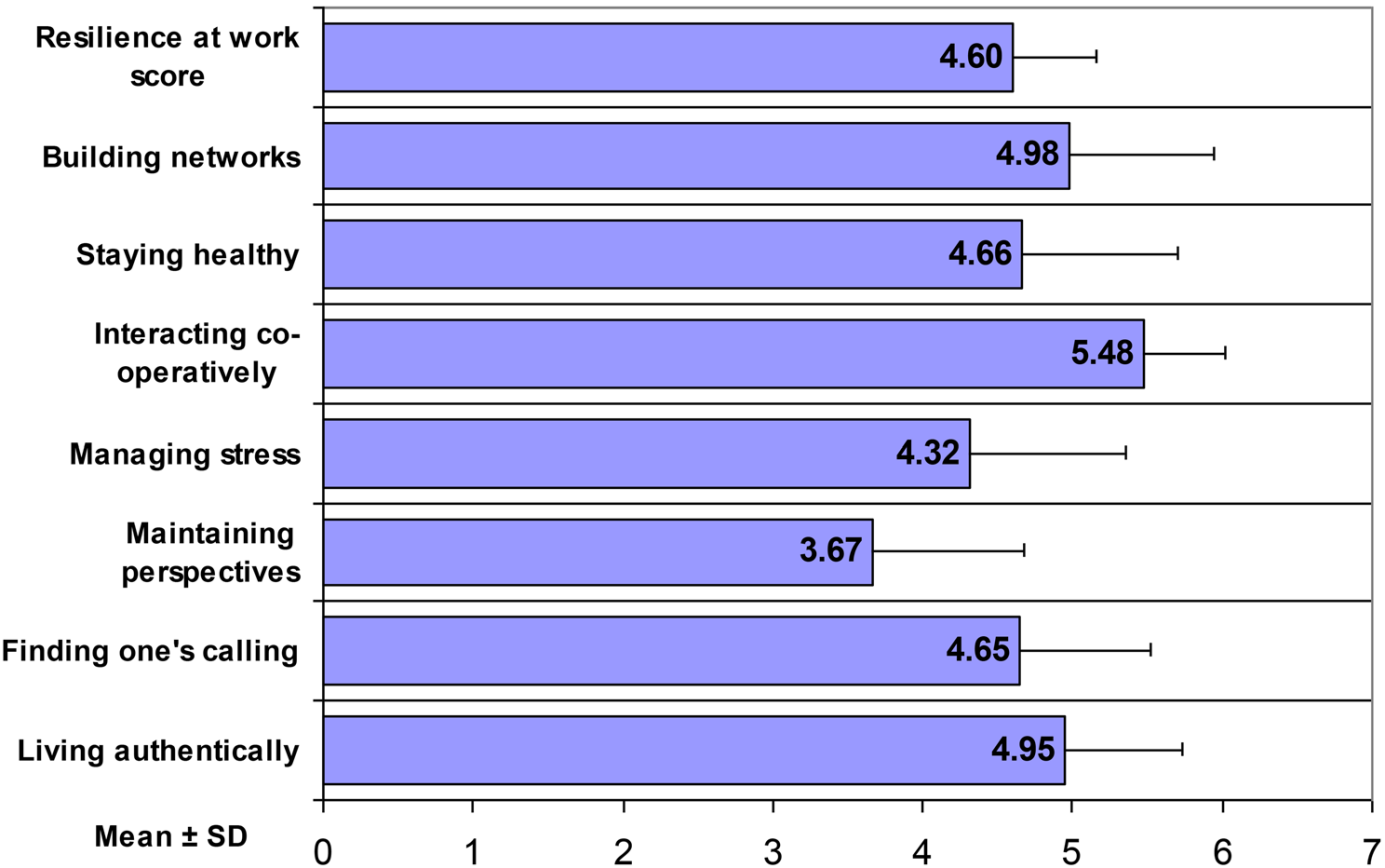
Resilience at work scores of mental health nurses across several dimensions

Figure 3 presents the mean scores for workplace resilience across its seven component dimensions. The overall mean resilience at work score was 4.60 ± 0.56, indicating relatively high resilience levels among mental health nurses. Dimensional analysis revealed substantial variation across components.

Interacting cooperatively demonstrated the highest mean score (5.48 ± 0.54), indicating strong collaborative behaviors and supportiveness among colleagues. Building networks (4.98 ± 0.96) and living authentically (4.95 ± 0.78) also exhibited relatively high scores, suggesting that nurses maintain meaningful professional relationships and remain true to their personal values in the workplace. Finding one’s calling showed a mean score of 4.94 ± 0.84, while staying healthy demonstrated a mean score of 4.69 ± 0.90.

Managing stress yielded a mean score of 4.41 ± 0.86, indicating moderate stress management capabilities. Notably, maintaining perspective demonstrated the lowest mean score among all dimensions (3.67 ± 1.00).

**Fig. 4:**
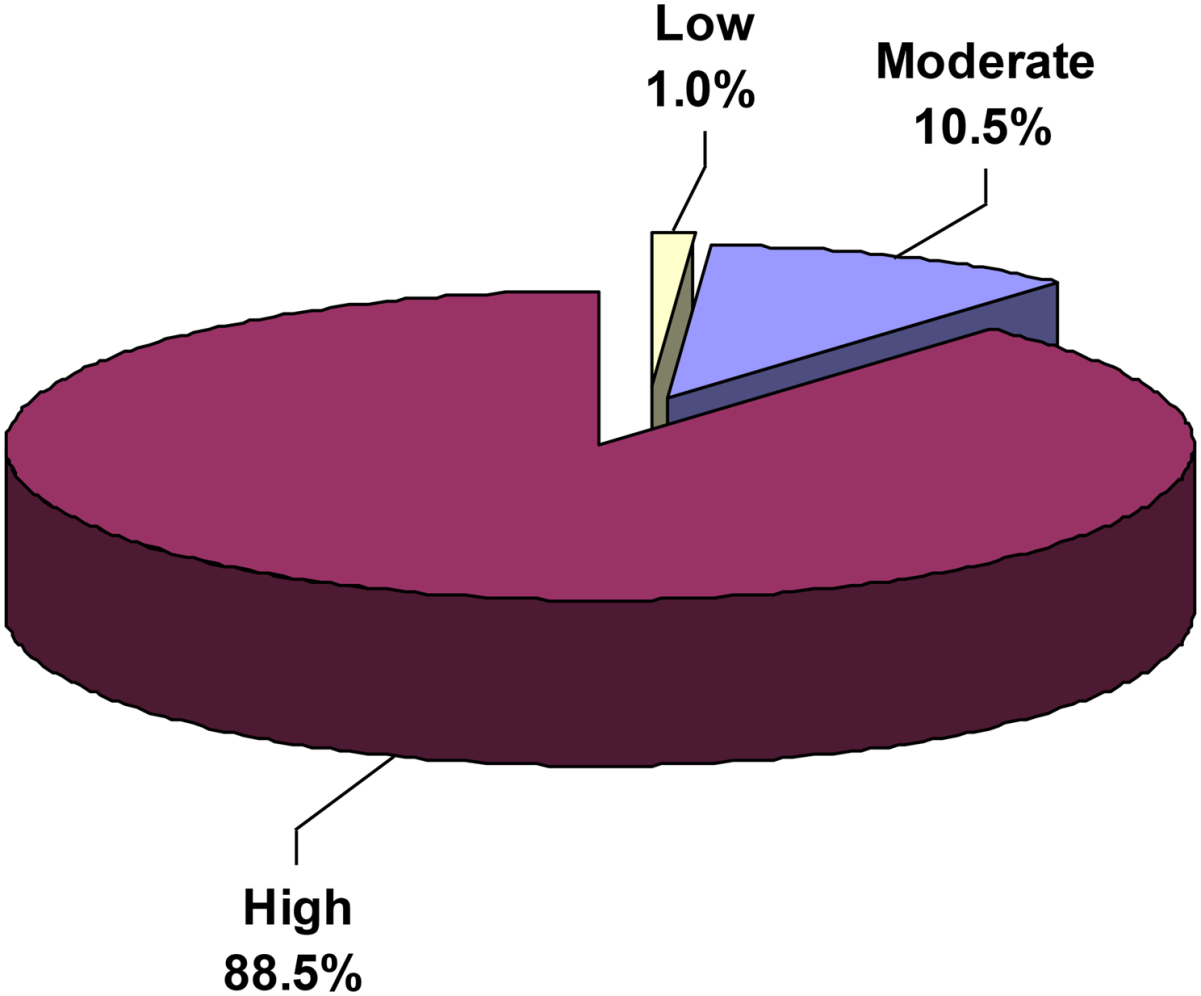
Level of resilience at work among mental health nurses

Figure 4 reveals the distribution of workplace resilience levels across the sample. The vast majority of nurses (88.5%) demonstrated high levels of workplace resilience (scores > 81), while 10.5% exhibited moderate levels (scores 61–81), and only 1% demonstrated low levels of workplace resilience (scores < 61).

### Predictors of psychological well-being

The multivariate linear regression analysis model examining predictors of psychological well-being (see Table 2). The analysis revealed that three variables emerged as significant predictors of higher psychological well-being scores. Male gender was significantly associated with higher psychological well-being (B = 0.294, 95% CI: 0.153–0.435, *p* < 0.001), indicating that male nurses reported better psychological well-being compared to their female counterparts. Employment in university hospitals was also a significant predictor of psychological well-being (B = 0.203, 95% CI: 0.066–0.340, *p* = 0.004). Additionally, higher socioeconomic status significantly predicted better psychological well-being (B = 0.242, 95% CI: 0.074–0.410, *p* = 0.005).

**Table 2:** Predictors of psychological well-being among mental health nurses

	Unstandardized coefficients		Standardized coefficients	t	*P*-value	95.0% CI for B	
	B	SE	Beta			Lower	Upper
Age (years)	0.015	0.018	0.278	0.821	0.413	-0.021	0.050
Gender (Male)	0.282	0.065	0.294	4.327	< 0.001*	0.153	0.410
Residence (Urban)	0.094	0.066	0.100	1.426	0.156	-0.036	0.225
Educational level	-0.034	0.075	-0.060	-0.458	0.648	-0.182	0.113
Religion (Muslim)	-0.018	0.086	-0.014	-0.205	0.838	-0.188	0.153
Marital status (Married)	0.085	0.085	0.068	0.996	0.321	-0.083	0.253
Years of experience	-0.016	0.018	-0.354	-0.913	0.362	-0.051	0.019
Place of work (AUH)	0.213	0.072	0.203	2.943	0.004*	0.070	0.356
Socioeconomic score	0.019	0.007	0.242	2.827	0.005*	0.006	0.032

R² = 0.188; Adjusted R² = 0.150

### Predictors of workplace resilience

The multivariate linear regression analysis examining predictors of workplace resilience (see Table 3). Two variables emerged as significant predictors of higher workplace resilience scores. Employment in university hospitals was significantly associated with enhanced resilience (B = 0.177, 95% CI: 0.034–0.320, *p* = 0.016). Higher socioeconomic status was also a significant predictor (B = 0.322, 95% CI: 0.150–0.494, *p* = 0.0001).

**Table 3:** Predictors of workplace resilience among mental health nurses

	Unstandardized Coefficients		Standardized coefficients	t	*P*-value	95.0% CI for B	
	B	SE	Beta			Lower	Upper
Age (years)	0.006	0.023	0.088	0.245	0.807	-0.039	0.051
Gender (Male)	0.095	0.083	0.083	1.147	0.253	-0.069	0.259
Residence (Urban)	0.046	0.084	0.041	0.547	0.585	-0.120	0.213
Educational level	-0.131	0.096	-0.190	-1.371	0.172	-0.320	0.057
Religion (Muslim)	0.118	0.110	0.076	1.068	0.287	-0.100	0.335
Marital status (Married)	-0.028	0.109	-0.019	-0.255	0.799	-0.242	0.187
Years of experience	-0.006	0.023	-0.108	-0.262	0.794	-0.050	0.039
Place of work (AUH)	0.224	0.092	0.177	2.426	0.016*	0.042	0.406
Socioeconomic score	0.030	0.008	0.322	3.555	< 0.001*	0.013	0.046

R² = 0.087; Adjusted R² = 0.044

## Discussion

This study assessed the psychological well-being and workplace resilience of nurses employed in mental health hospitals in Assiut District and investigated factors associated with these outcomes. In Egypt, no previous study has examined this issue among psychiatric nurses specifically. This study found that nearly half of mental health nurses reported favorable psychological well-being. This finding aligns with previous studies conducted by Delgado et al. and Foster et al. [1, 2]. This is because these studies also focused on mental health nurses working in high-emotional-demand environments with subsequent exposure to patient aggression. However, the finding of the present study is lower than that reported by Eunhye and Sunhee [21]. This may be attributable to differences in healthcare system structure, including stronger institutional support and greater availability of mental health resources in high-income settings. Deterioration of psychological well-being may occur in the absence of effective organizational socialization, as nurses must adapt not only to their professional role but also to the structural hospital environment [22]. The present study revealed that personal growth (the sense of accomplishment derived from realizing one’s potential and embracing new challenges) and environmental mastery (successfully adapting to new environments and effectively managing complex situations) demonstrated high scores. However, purpose in life (awareness of meaning, value, and direction in life) exhibited a low score. This pattern suggests unmet higher-order needs among nurses who have successfully adapted to the hospital environment and satisfied their basic security, safety, and social needs. According to Benner’s theory, nursing expertise involves seeking holistic understanding and situational perception [23]. While professional proficiency enhances decision-making ability through holistic understanding, this study revealed declining autonomy (independence from social conventions and others’ standards). Benner’s theory stated that the development of nursing expertise is accompanied by enhanced clinical judgment, intuitive decision-making, and a deeper professional identity. As nurses progress toward expert practice, they are expected to demonstrate greater autonomy in clinical reasoning and patient care. However, the findings of the present study suggest that professional expertise does not necessarily translate into perceived autonomy. This apparent decline in autonomy may be explained by organizational and structural constraints within psychiatric hospital settings, such as rigid hierarchical decision-making, physician-dominated care models, standardized protocols, and limited participation of nurses in organizational governance. Consequently, although experienced mental health nurses possess advanced clinical competence and a strong professional identity, their ability to exercise independent decision-making may be restricted by institutional factors rather than individual capability.

Gender differences persist across various background characteristics, generally disfavoring females. Previous research has shown that females tend to report poorer health status compared to males [24–26]. This study demonstrates higher prevalence of psychological well-being among males, consistent with other studies [27–29]. This can be explained by the fact that women are often socialized to assume caregiving role both at work and within the family, which may intensify their overload, particularly in mentally healthcare settings such as psychiatric hospitals. This dual burden may place female nurses at greater risk of emotional exhaustion and reduced psychological well-being. However, male nurses may experience fewer expectations, allowing greater psychological well-being. Research has demonstrated the importance of economic resources in maintaining psychological health and enhanced subjective well-being among adults [30]. This was confirmed in the present study, as participants with higher socioeconomic status reported superior psychological well-being. Additionally, this study found higher psychological well-being among participants employed in university hospitals compared to those in Ministry of Health mental health hospitals, potentially attributable to greater specialist availability in university hospitals with consequent reduced workload. In addition, higher psychological well-being and workplace resilience among nurses employed in university hospitals may be partly explained by differences in workload characteristics between university and Ministry of Health hospitals. University hospitals typically benefit from more favorable nurse-to-patient ratios, greater predominance of multidisciplinary teams, and obvious role delineation, which collectively reduces individual workload burden. However, Ministry of Health mental health hospitals often face physicians’ shortages, higher patient volumes, and limited material and human resources, resulting in increased work intensity and prolonged exposure to occupational stressors.

High resilience refers to a nurse’s ability to effectively cope with stress, adapt to challenges, and maintain focus and performance in demanding clinical situations. This means that nurses with high resilience can manage heavy workloads, recover quickly from setbacks, communicate effectively under pressure, and provide consistent, high-quality patient care even in stressful or rapidly changing environments. Workplace resilience is essential for managing the demands of nursing practice [31]. Research has shown that personal and work-related factors are associated with nurse resilience, with measurement varying significantly and affecting consistency in identifying influencing factors [31].

This study demonstrated high levels of workplace resilience among nurses employed in mental health hospitals in Upper Egypt. Egyptian nurses exhibited higher resilience levels than Jordanian [32], Greek [33], and American [34] nurses. Heterogeneity in workplace resilience values across healthcare organizations may relate to different cultural contexts, conditions, and organizational circumstances [35]. This underscores the need to develop tailored strategies for enhancing workplace resilience among nursing staff and maintaining standards that ensure the well-being of staff, patients, and other healthcare service users within healthcare organizations.

Regarding resilience at work dimensions, the highest mean subscale scores were interacting cooperatively, building networks, and living authentically, consistent with findings from Carpio et al. [34]. The interacting cooperatively subscale mean score suggests that mental health nurses demonstrate responsiveness to feedback and interaction while providing support to colleagues. The living authentic score reveals that mental health nurses maintain strong personal values, utilize their personal strengths to benefit patients, and demonstrate high empathy levels. The maintaining perspective subscale exhibited the lowest mean score, suggesting that negative workplace influences affect nurses’ perspectives, and when workplace problems arise, mental health nurses tend to experience worry rather than feeling motivated to seek solutions. Furthermore, low maintaining perspective subscale among studied nurses can be attributed to chronic exposure to mental health crises, emotional fatigue and absence of psychological support frameworks.

Socioeconomic status appears to play an important role in determining positive psychological states such as resilience, hope, optimism, and happiness [36]. Socioeconomic level is also frequently recognized as a determinant of resilience [36]. This study demonstrated that participants with higher socioeconomic status exhibited higher resilience scores, consistent with other studies [36, 37]. Furthermore, this study found higher resilience among participants employed in university hospitals compared to those in Ministry of Health mental health hospitals. This may be explained by the higher psychological well-being observed among university hospital participants in this study, with consequent elevated resilience levels. Furthermore, variations in training availability may help explain the higher levels of psychological well-being and workplace resilience observed among nurses working in university hospitals. These settings generally offer greater access to continuing education, in-service training, clinical supervision, and opportunities to engage in workshops, conferences, and research activities. Such professional development initiatives strengthen clinical competence, decision-making abilities, and professional self-efficacy, which are closely associated with enhanced autonomy, environmental mastery, and personal growth [39].

In addition, differences in access to organizational and clinical resources may further account for the disparities in psychological well-being and workplace resilience observed between nurses working in university hospitals and those in Ministry of Health mental health hospitals. University hospitals are generally better resourced, with greater availability of medical supplies, therapeutic equipment, information systems, and specialized support services, which facilitate efficient care delivery and reduce work-related frustration [38]. This study has some limitations. First, its cross-sectional design prevents the establishment of causal relationships between independent and dependent variables. Second, the use of convenience sampling and the assessment of mental health nurses at a single time point within a specific geographic region limit the generalizability of the findings. Additionally, the study may be subject to bias, including social desirability bias related to interview-based data collection and self-reporting bias. However, a key strength of this study is the utilization of standardized measurement instruments that have been widely used in international research, allowing for cultural comparability of psychological well-being and workplace resilience. Future research incorporating qualitative methods may provide a deeper understanding of nurses’ experiences and contextual factors influencing psychological well-being and workplace resilience. Evaluation of the effectiveness of targeted interventions, such as resilience training programs, peer-support initiatives, or organizational policy changes, in improving nurses’ psychological well-being should be considered in future research.

## Conclusion

The psychological well-being of mental health nurses was moderate (51%) or favorable (49%). Autonomy and purpose in life dimensions were lower compared to other psychological well-being dimensions. Additionally, 88.5% of nurses demonstrated high levels of workplace resilience. Interacting cooperatively exhibited the highest mean score, while maintaining perspective demonstrated the lowest score. Significant predictors of both psychological well-being and workplace resilience included employment in university hospitals and higher socioeconomic status. Male gender emerged as a significant predictor of psychological well-being. The implementation of regular stress management training programs that emphasize adaptive coping strategies, emotional regulation, and effective workload management may help alleviate reduced psychological well-being. In addition, resilience-building workshops that strengthen problem-solving skills, foster perspective-taking, and enhance peer support could further improve nurses’ capacity to cope with the demands of psychiatric care settings. Moreover, leadership development programs for nurse managers are recommended to promote supportive supervision, equitable workload distribution, and inclusive decision-making, all of which are critical for enhancing nurses’ autonomy and sense of purpose at work.

## Data Availability

The datasets used and analyzed during this study are available from the corresponding author upon reasonable request.

## Abbreviations

SD: Standard Deviation
